# Changes in haemodynamics during single lung transplantation under venovenous extracorporeal membrane oxygenation

**DOI:** 10.1093/icvts/ivac101

**Published:** 2022-04-21

**Authors:** Hisashi Oishi, Yasushi Matsuda, Yutaka Ejima, Hiroaki Toyama, Takashi Hirama, Tatsuaki Watanabe, Yui Watanabe, Hiromichi Niikawa, Masafumi Noda, Yoshinori Okada

**Affiliations:** 1 Department of Thoracic Surgery, Institute of Development, Ageing and Cancer, Tohoku University, Sendai, Japan; 2 Department of Thoracic Surgery, Fujita Health University School of Medicine, Toyoake, Japan; 3 Department of Anesthesiology, Tohoku University Hospital, Sendai, Japan

**Keywords:** Lung transplantation, Extracorporeal membrane oxygenation, Pulmonary hypertension, Pulmonary arterial pressure

## Abstract

**OBJECTIVES:**

The objective of the present study was to examine the effect of venovenous (VV) extracorporeal membrane oxygenation (ECMO) use on the haemodynamics during single lung transplantation (SLT) and postoperative course.

**METHODS:**

Forty-seven patients who underwent SLT for end-stage lung diseases in our lung transplant centre between January 2010 and December 2019 were included in this study. The recipients were divided into 3 groups according to the type of intraoperative ECMO. No type of ECMO was intra-operatively used in the patients of the no use of ECMO (NO ECMO) group. The patients in the venoarterial (VA) and VV ECMO groups were put on VA and VV ECMO during the surgery, respectively. The data were compared among the 3 groups.

**RESULTS:**

There were 13 SLT cases in the NO ECMO group, 23 SLT cases in the VA ECMO group and 11 SLT cases in the VV ECMO group. Re-exploration for bleeding was performed in 3 (13.0%) recipients in the VA ECMO group. No recipients required re-exploration in the other groups. In the NO ECMO group, systolic pulmonary arterial pressure (PAP) was significantly elevated during the main pulmonary artery clamp on the SLT side and it was decreased in the VA ECMO group because of the bypass flow. Interestingly, systolic PAP was significantly decreased in the VV ECMO group as well.

**CONCLUSIONS:**

VV ECMO decreases the PAP during SLT, which could be a choice for extracorporeal life support during lung transplant surgery for patients, even those with pulmonary hypertension.

**Figure:**
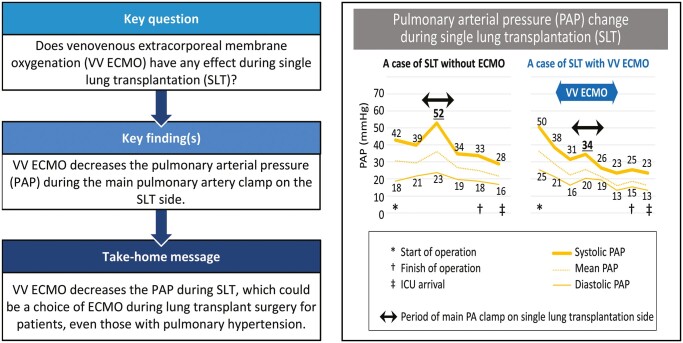

## INTRODUCTION

Lung transplantation is now an established treatment for patients with end-stage lung diseases. Varying widely depending on the institution, 27–40% [1–3] of recipients require some kind of extracorporeal life support (ECLS) during lung transplant surgery. Cardiopulmonary bypass (CPB) had been traditionally used for intraoperative cardiorespiratory support during single and bilateral lung transplantations for many years. Recently, more lung transplant centres have started using extracorporeal membrane oxygenation (ECMO) and the number of lung transplantations performed under ECMO has been dramatically increasing [4]. Machuca *et al.* [5] reported that ECMO has significant advantages over CPB for both intraoperative and early post-transplant outcomes. More recently, there are 2 options as an intraoperative ECMO for lung transplantation—venoarterial (VA) or venovenous (VV) ECMO.

In the field of lung transplantation, VV ECMO is an effective tool to bridge patients on the lung transplant waitlist for lung transplantation [6]. VV ECMO has also been used as an intraoperative ECLS for single lung transplantation (SLT) in recent years [4]. Ius *et al.* reported that, among 311 recipients who underwent lung transplantation under ECMO support, the ECMO configuration was VV in 34 recipients, VA in 271 recipients and veno-veno-arterial in 6 recipients [3].

We had previously been utilizing VA ECMO when an intraoperative ECLS was needed for SLT. We had believed that haemodynamic support was required in every SLT case, along with gas exchange support. The reason for that was based on the assumption that an elevation of pulmonary arterial pressure (PAP) mainly resulted from a reduction of the capillary bed in the lung during the time period between the extraction of the native lung and the reperfusion of the lung graft. In addition to this mechanism, the hypoxic pulmonary vasoconstriction (HPV) response in the lung is thought to be related to the PAP elevation. HPV is maximal when the mixed venous oxygen saturation (SvO_2_) is normal and is decreased by either high or low SvO_2_ [7]. A significant elevation of SvO_2_ during VV ECMO was reported to potentially relieve HPV, reducing pulmonary pressure [8]. In addition, Morimont *et al.* [9] reported that VV extracorporeal CO_2_ removal ameliorated pulmonary hypertension (PH) and improved the right ventricular function in a porcine acute respiratory distress syndrome model.

The objective of the present study was to retrospectively examine the effect of VV ECMO use on the haemodynamics during SLT and the postoperative course. We especially investigated the change of PAP during SLT and the early post-transplant outcome.

## PATIENTS AND METHODS

### Ethical statement

The institutional review board of Tohoku University Hospital approved this retrospective study (No. 2019-1-766) and waived the requirement for informed consent. Using the donor data for this research was approved by the institutional review board of Japan Organ Transplant Network.

### Patients, data collection and study groups

We reviewed the records of 51 patients who underwent SLT for end-stage lung diseases in our lung transplant centre between January 2010 and December 2019. (There were 43 cases of bilateral lung transplantation in our centre during the study period.) Patients with a past history of pleurodesis due to pneumothorax are indicated for lung transplantation in our centre. Cases without detailed PAP data and retransplantations were excluded from this study and, ultimately, 47 patients were included. There was no patient in the present study who had been on ECMO before lung transplantation. The recipients were divided into 3 groups according to the use and type of intraoperative ECMO. No type of ECMO was intraoperatively used in the patients of the no use of ECMO (NO ECMO) group. The patients in the VA and VV ECMO groups were put on VA and VV ECMO during the surgery, respectively. The data were compared among the 3 groups.

### ECMO induction procedure and intraoperative management

We had utilized VA ECMO as an ECLS during SLT before 2015 and started using VV ECMO thereafter in cases that did not show a dominant perfusion ratio on the SLT side (>80%) by pretransplant lung perfusion scintigraphy or severe PH [systolic PAP (sPAP) of >70 mmHg]. We preoperatively decided the type of ECMO to use. Furthermore, immediately after the induction of general anaesthesia, percutaneous insertion of a Swan-Ganz catheter through the right or left internal jugular vein was performed and the monitoring of PAP was initiated. We also inserted a probe for the transoesophageal echocardiogram into the oesophagus and continued viewing the heart throughout the surgery, especially for the assessment of the right ventricle. We prepared to switch VA to VV ECMO support for circulatory support and the operation (OP) was started.

In every SLT surgery, we clamped the main pulmonary artery (PA) on the SLT side prior to the extraction of the native lung to determine whether the patient’s oxygenation and circulatory condition were tolerable. At that time, we initiated nitric oxide inhalation in all cases to alleviate PH.

For the establishment of VA ECMO, we chose the patient’s right or left femoral artery (inflow) and vein (outflow) for the cannulation site: peripheral VA ECMO. Or, we chose the ascending aorta and right or left femoral vein: central VA ECMO. When we used the femoral artery for the inflow, we inserted the inflow cannula into the vascular graft that was sutured to the femoral artery instead of directly inserting the cannula into it. The blood flow of VA ECMO was initially set at 50% of the estimated cardiac output of the recipient and adjusted according to the result of the arterial blood gas analysis and the haemodynamic status. Heparin was administered to adjust the activated clotting time to 180–200 s with VA ECMO. We reperfused the lung graft very slowly after the anastomosis by gradually reducing the blood flow of the VA ECMO. When we weaned the patient from the VA ECMO, we sutured the decannulation site of the femoral vein and ligated the vascular graft sewn to the femoral artery (peripheral VA ECMO). Or, we sutured the decannulation site of the ascending artery (central VA ECMO).

We established VV ECMO by using right and left femoral veins for cannulation sites (inflow and outflow). The cannulations were percutaneously performed under ultrasound guidance. VV ECMO was started at a blood flow of 50% of the estimated cardiac output of the recipient. The administration of heparin was controlled to target activated clotting time from 160 to 180 s. We reperfused the lung graft very slowly after the anastomosis by unclamping the PA, taking 10 min. When we weaned the patient from the VV ECMO, the haemostasis of the decannulation sites was performed by compression.

## STATISTICAL ANALYSIS

Continuous data are expressed as median with range. Categorical variables are presented as number and percentage. Comparisons of continuous variables among the 3 groups were made by one-way analysis of variance followed by a *post hoc* analysis using Bonferroni methods. Repeated measures two-way analysis of variance with *post hoc* Bonferroni methods were used for comparisons of the sPAP and systolic blood pressure (sBP). Categorical variables were compared using the χ^2^ test or Fisher’s exact test. Differences were considered significant at the *P *<* *0.05 level. Statistical analyses were performed using Prism 5 (GraphPad Software Inc, La Jolla, Calif) and SPSS Statistics 21.0 for Windows (IBM Corporation, Chicago, IL, USA).

## RESULTS

### Extracorporeal membrane oxygenation use during single lung transplantation at Tohoku University Hospital

**Figure 1:**
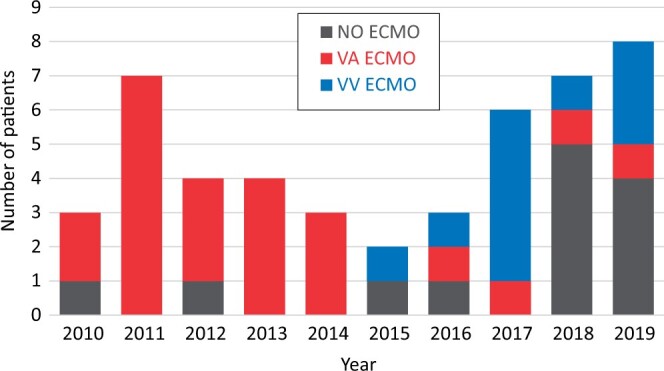
Number of extracorporeal membrane oxygenation precedures during single lung transplantation at Tohoku University Hospital. The numbers of patients without extracorporeal membrane oxygenation and those with venovenous extracorporeal membrane oxygenation increased during the last 5 years. On the other hand, the number of patients who underwent lung transplantation under veno-arterial extracorporeal membrane oxygenation support has decreased since 2014.

There were 13 SLT cases in the NO ECMO group, 23 SLT cases in the VA ECMO group (central VA ECMO, 2 cases; peripheral VA ECMO, 21 cases) and 11 SLT cases in the VV ECMO group. Figure 1 shows the ECMO type during lung transplantations at each year in the study period. We started utilizing VV ECMO in 2015, and the number of patients with VV ECMO increased over the past 4 years. On the other hand, the number of patients who underwent lung transplantation under VA ECMO has decreased since 2015.

### Demographics of the donors and pretransplant demographics of the recipients

Table 1 shows the demographics of the donors. There was no significant difference in any parameters among the 3 groups. Table 2 shows the pretransplant demographics of the recipients. Recipient age, gender, height, weight, body mass index and transplant side were similar among the 3 groups. The number of patients with lymphangioleiomyomatosis (LAM) in the VA ECMO group tended to be greater than that in the other 2 groups and, therefore, there were more female patients in the VA ECMO group; however, the differences were not statistically significant. The perfusion ratio on the SLT side in lung perfusion scintigraphy was comparable among the 3 groups. The left ventricular ejection fraction measured by echocardiography was also similar among the 3 groups. The sPAP in the VV ECMO group showed a tendency to be higher than that in the other 2 groups; however, the difference was not statistically significant.

**Table 1:** Demographics of the donors

	NO ECMO group	VA ECMO group	VV ECMO group	*P*-Value
	(*N* = 13)	(*N* = 23)	(*N* = 11)	
Donor age (years)	44 (11–64)	47 (26–68)	43 (18–67)	0.18
Donor gender (M/F)	6/7	6/17	6/5	0.22
Donor height (cm)	162 (140–178)	159 (140–176)	165 (154–186)	0.23
Donor weight (kg)	60 (43.0–87.7)	57.6 (42.0–75.0)	61.5 (47.3–90.5)	0.23
Donor BMI (kg/m^2^)	23.5 (18.5–42.3)	21.5 (16.1–28.6)	22.4 (17.4–28.7)	0.20
Smoking history (pack-years)				0.75
None	6 (46.2)	13 (56.5)	4 (36.4)	
0–20	3 (23.1)	6 (26.1)	3 (27.3)	
≤20	4 (30.8)	4 (17.4)	4 (36.4)	
PaO_2_/FiO_2_ (mmHg)	466 (229–650)	519 (273–635)	519 (336–606)	0.24
Cause of brain death				0.92
Cerebrovascular accident	8 (61.5)	14 (60.9)	7 (63.6)	
Brain ischaemia	4 (30.8)	6 (26.1)	2 (18.2)	
Others	1 (7.7)	3 (13.0)	2 (18.2)	

Values are expressed as medians (range) or number (%).

BMI: body mass index; ECMO: extracorporeal membrane oxygenation; F: female; M: male; NO ECMO: no use of ECMO; VA: venoarterial; VV: venovenous; PaO_2_/FiO_2_: arterial oxygen partial pressure/fraction of inspired oxygen concentration ratio.

**Table 2:** Pre-transplant demographics of the recipients

	NO ECMO group	VA ECMO group	VV ECMO group	*P*-Value
	(*N* = 13)	(*N* = 23)	(*N* = 11)	
Recipient age (years)	48 (29–62)	46 (35–61)	49 (23–61)	0.72
Recipient gender (M/F)	7/6	5/18	6/5	0.07
Recipient height (cm)	165 (153–175)	160 (147–174)	160 (149–172)	0.54
Recipient weight (kg)	54.2 (33.3–77.6)	47.4 (32.6–72.9)	58.7 (26.3–93.3)	0.40
Recipient BMI (kg/m^2^)	19.6 (12.4–30.4)	16.8 (13.4–31.1)	20.9 (11.8–31.7)	0.67
Transplant side (left/right)	6/7	10/13	9/2	0.09
Indication				0.33
LAM	4 (30.8)	13 (56.5)	3 (27.3)	
IPF	2 (15.4)	4 (17.4)	4 (36.4)	
COPD	4 (30.8)	1 (4.3)	1 (9.1)	
CTD-ILD	2 (15.4)	2 (8.7)	2 (18.2)	
Others	1 (7.7)	3 (13.0)	1 (9.1)	
Perfusion ratio on SLT side (%)	49.9 (2.1–74.3)	49.0 (27.3–88.5)	42.9 (22.9–59.0)	0.48
LVEF by echocardiography (%)	64 (43–71)	63 (44–79)	62 (58–74)	0.65
Preoperative sPAP (mmHg)	43 (27–52)	41 (25–88)	48 (31–78)	0.34

Values are expressed as medians (range) or number (%).

BMI: body mass index; COPD: chronic obstructive pulmonary; CTD-ILD: connective tissue disease-associated interstitial lung disease; ECMO: extracorporeal membrane oxygenation; F: female; IPF: idiopathic pulmonary fibrosis; LAM: lymphangioleiomyomatosis; LVEF: left ventricular ejection fraction; M: male; NO ECMO: no use of ECMO; SLT: single lung transplantation; sPAP: systolic pulmonary arterial pressure; VA: venoarterial; VV: venovenous.

### Operative and postoperative characteristics

Table 3 shows the operative and postoperative characteristics. There was no significant difference in the operative time, cold ischaemic time or intraoperative blood loss among the 3 groups. The intraoperative ECMO time was significantly longer in the VV ECMO group than in the VA ECMO group. In the arterial blood gas analysis during the main PA clamp on the SLT side, the pH level was significantly lower in the NO ECMO group compared to the VA and VV groups (*P *<* *0.01). The pCO_2_ level was significantly higher in the NO ECMO group compared to the VA and VV groups (*P *<* *0.01). The pO_2_ level tended to be lower in the NO ECMO group than in the other groups, but the difference was not statistically significant (*P *=* *0.63).

**Table 3:** Operative and postoperative characteristics

	NO ECMO group	VA ECMO group	VV ECMO group	*P*-Value
	(*N* = 13)	(*N* = 23)	(*N* = 11)	
Operative time (min)	389 (298–452)	410 (243–646)	440 (356–504)	0.28
Cold ischaemic time (min)	457 (361–546)	454 (361–579)	493 (400–656)	0.14
Intra-OP ECMO time (min)	N/A	412 (147–697)	488 (380–572)	0.002^a^
Intra-OP blood loss (ml)	307 (79–2122)	848 (137–6468)	1079 (177–6228)	0.13
Arterial blood gas analysis (during PA clamp on SLT side)				
pH	7.22 (7.09–7.29)	7.38 (7.17–7.54)	7.39 (7.34–7.43)	<0.01^b^
pCO_2_ (mmHg)	63.9 (52.8–107)	43.4 (32.9–65.3)	43.2 (28.3–50.6)	<0.01^c^
pO_2_ (mmHg)	181 (80–513)	267 (118–459)	212 (98–509)	0.63
ECMO on ICU arrival	0 (0)	7 (30.4)	3 (27.3)	
Primary graft dysfunction^d^	1 (7.7)	9 (39.1)	4 (36.4)	0.12
Re-exploration for bleeding	0 (0)	3 (13.0)	0 (0)	
Postoperative CVVH	0 (0)	3 (13.0)	2 (18.2)	
Intra-operative blood transfusion				
RBC (ml)	0 (0–2800)	840 (0–2800)	560 (0–1960)	0.09
FFP (ml)	0 (0–2800)	480 (0–3360)	480 (0–1680)	0.07
Platelet (ml)	0 (0–0)	0 (0–720)	0 (0–240)	0.02^e^
Postoperative blood transfusion^f^				
RBC (ml)	0 (0–2800)	280 (0–1960)	280 (0–840)	<0.01^g^
FFP (ml)	0 (0–0)	240 (0–720)	240 (0–480)	0.05
Platelet (ml)	0 (0–480)	0 (0–480)	240 (0–240)	0.17

Values are expressed as median (range) or number (%).

a Comparison was performed between VA ECMO and VV ECMO group.

b NO ECMO versus VA ECMO and NO ECMO versus VV ECMO are significant.

c NO ECMO versus VA ECMO and NO ECMO versus VV ECMO are significant.

d Primary graft dysfunction was defined as any International Society for Heart and Lung Transplantation Grade 3 primary graft dysfunction within 72 h of reperfusion.

e NO ECMO versus VA ECMO is significant.

f The amount of transfusion of each blood product within 72 h.

g NO ECMO versus VA ECMO and NO ECMO versus VV ECMO are significant.

CVVH: continuous veno-venous hemofiltration; ECMO: extracorporeal membrane oxygenation; FFP: fresh frozen plasma; NO ECMO: no use of ECMO; OP: operative; PA: pulmonary artery; RBC: red blood cell; SLR: single lung transplantation; VA: venoarterial; VV: venovenous.

In the NO ECMO group, no recipient was on ECMO at the time of intensive care unit (ICU) arrival. There were 7 (30.4%) and 3 (27.3%) recipients on ECMO on ICU arrival in the VA and VV ECMO groups, respectively. The patients were all weaned off ECMO within 5 days in the VA and VV ECMO groups, with the exception of a 90-day mortality case with VV ECMO. Primary graft dysfunction (PGD) was developed in 1 (7.7%), 9 (39.1%) and 4 (36.4%) in the NO, VA and VV ECMO groups, respectively. There was no significant difference in the incidence of PGD among the 3 groups. PGD was defined as any International Society for Heart and Lung Transplantation Grade 3 PGD [10] within 72 h of reperfusion. Re-exploration for bleeding was performed in 3 (13.0%) recipients in the VA ECMO group. On the other hand, no recipients needed re-exploration in the other groups. Continuous venovenous hemofiltration was required in 3 (13.0%) and 2 (18.2%) recipients in the VA and VV ECMO groups, respectively.

**Figure 2:**
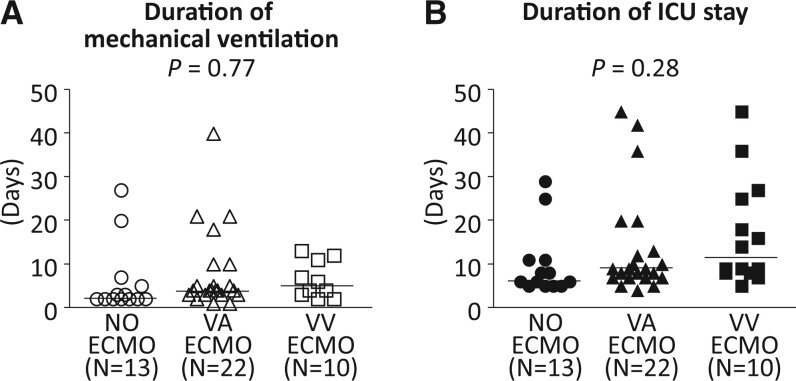
Duration of mechanical ventilation and ICU stay after single lung transplantation. (**A**) The median duration of mechanical ventilation (days) was 2 (2–27) in the no use of extracorporeal membrane oxygenation group, 4 (1–40) in the venoarterial extracorporeal membrane oxygenation group and 5 (2–13) in the venovenous extracorporeal membrane oxygenation group. There was no significant difference (*P* = 0.77) among the 3 groups. (**B**) The median ICU stay period (days) was 6 (5–29) in the no use of extracorporeal membrane oxygenation group, 9 (4–45) in the venoarterial extracorporeal membrane oxygenation group and 11.5 (5–27) in the venovenous extracorporeal membrane oxygenation group. There was no significant difference (*P* = 0.28) among the 3 groups.

Figure 2A and B shows the duration of mechanical ventilation and ICU stay, respectively. There was no significant difference in the duration of mechanical ventilation (*P *=* *0.77) or the ICU stay period (*P *=* *0.28) among the 3 groups.

### Operative and postoperative blood transfusion

There was no significant difference in the amount of intraoperative red blood cells (RBCs) and fresh frozen plasma transfusion among the 3 groups. No recipient needed platelet transfusion in the NO ECMO group. The amount of intra-operative platelet transfusion was significantly greater in the VA ECMO group than that in the NO ECMO group (*P *=* *0.02). The amount of postoperative RBC transfusion was significantly smaller in the NO ECMO group compared to the VA and VV groups (*P *<* *0.01). There was no significant difference in the amount of postoperative RBC transfusion between the VA and VV groups. There was no significant difference in the amount of postoperative fresh frozen plasma and platelet transfusion among the 3 groups.

### Postoperative mortality

Supplementary Material, Table S1 shows the postoperative 30-day, 90-day and 1-year mortalities. There was no mortality at each time point in the NO ECMO group. There was a 90-day mortality in the VA and the VV ECMO groups. Similarly, there was a mortality in each of the VA and VV ECMO groups at postoperative 1 and 3 years.

### Changes in pulmonary arterial pressure during single lung transplantation surgery in a typical case from each group

**Figure 3:**
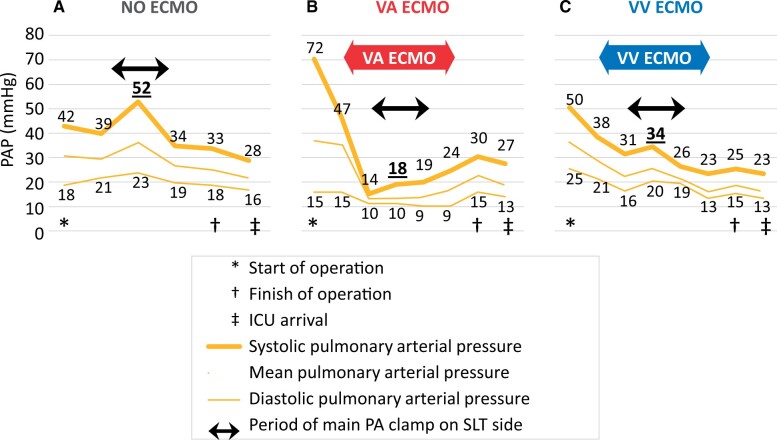
Pulmonary arterial pressure change during single lung transplantation surgery in a typical case from each group. (**A**) The patient in the no use of extracorporeal membrane oxygenation group underwent single lung transplantation for lymphangioleiomyomatosis and showed mild pulmonary hypertension at the start of operation. The systolic pulmonary arterial pressure of the patient was elevated up to 52 mmHg during the main pulmonary artery clamp for the extraction of the native lung and the anastomosis of the graft. (**B**) The patient in the venoarterial extracorporeal membrane oxygenation group received single lung transplantation for idiopathic pulmonary fibrosis and preoperatively showed severe pulmonary hypertension. We placed the patient on venoarterial extracorporeal membrane oxygenation and the systolic pulmonary arterial pressure was maintained at a low level during the main pulmonary artery clamping. (**C**) The patient in the venovenous extracorporeal membrane oxygenation group underwent single lung transplantation for lymphangioleiomyomatosis and showed mild pulmonary hypertension at the start of operation. The patient’s pulmonary arterial pressure decreased after the establishment of venovenous extracorporeal membrane oxygenation and did not elevate significantly even during the main pulmonary artery clamping on the single lung transplantation side.

Figure 3A–C shows the changes in PAP during SLT surgery in typical cases without ECMO, with VA or VV ECMO, respectively. The patient of the NO ECMO group underwent SLT for LAM and showed mild PH at the start of OP. The sPAP of the patient elevated up to 52 mmHg during the main PA clamping for the extraction of the native lung and the anastomosis of the graft. The patient of the VA ECMO group received SLT for idiopathic pulmonary fibrosis and preoperatively showed severe PH. We placed the patient on VA ECMO and the PAP was maintained at a low level during the main PA clamping. The patient of the VV ECMO group underwent SLT for LAM and showed mild PH at the start of OP. The patient’s PAP lowered after the establishment of VV ECMO and did not elevate significantly, even during the main PA clamping on the SLT side.

### Comparisons of systolic pulmonary arterial pressure and systolic blood pressure during single lung transplantation

**Figure 4:**
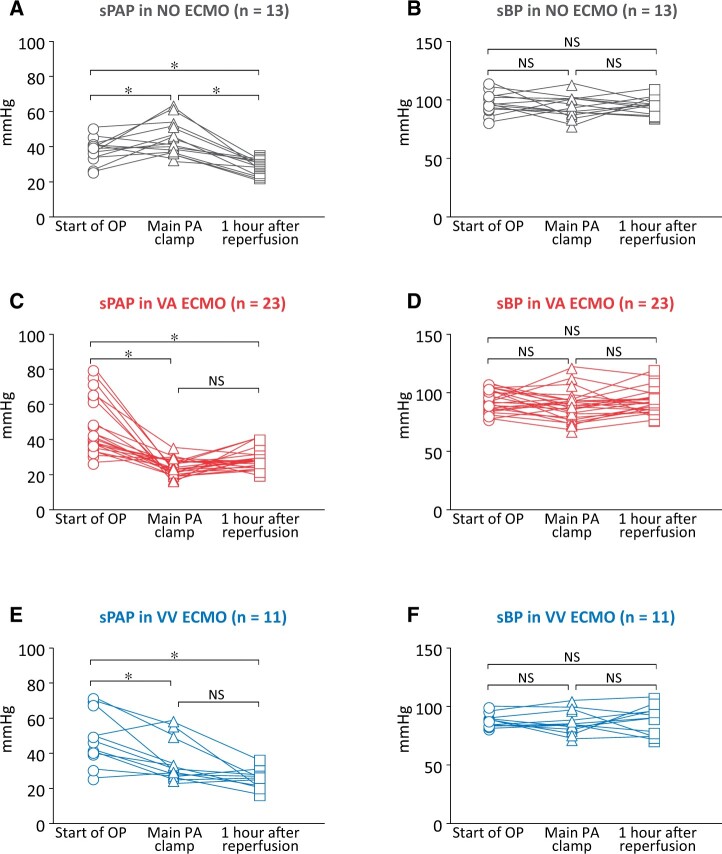
Comparisons of systolic pulmonary arterial pressure and systolic blood pressure during single lung transplantation. (**A** and **B**) In the no use of extracorporeal membrane oxygenation group, the median systolic pulmonary arterial pressure (mmHg) at the start of operation was 39 (26–51) and it significantly elevated to 42 (33–64) at the time of the main pulmonary artery clamping (*P* < 0.05). The systolic pulmonary arterial pressure significantly decreased to 31 (25–36) at 1 h after reperfusion (*P* < 0.05). The systolic blood pressure did not show significant change at the start of operation, the main pulmonary artery clamping and 1 h after reperfusion; 99 (84–118), 96 (81–117) and 99 (88–113). (**C** and **D**) In the venoarterial extracorporeal membrane oxygenation group, the median systolic pulmonary arterial pressure at the start of operation was 41 (27–80) and significantly decreased to 24 (17–36) at the time of the main pulmonary artery clamping (*P* < 0.05). It was maintained low at 29 (21–41) at 1 h after reperfusion. The systolic blood pressure showed no significant change at the start of operation, the main pulmonary artery clamping and 1 h after reperfusion; 93 (79–109), 90 (69–123) and 92 (78–121). (**E** and **F**) In the venovenous extracorporeal membrane oxygenation group, the median systolic pulmonary arterial pressure at the start of operation was 47 (26–72) and significantly decreased to 31 (25–59) at the time of the main pulmonary artery clamping (*P* < 0.05) and was maintained low at 28 (19–39) at 1 h after reperfusion. The systolic blood pressure did not show significant change at the time of start of operation, the main pulmonary artery clamping and 1 hour after reperfusion; 97 (90–109), 93 (81–114) and 99 (80–117). ∗*P* < 0.05. NS: no significance.

Figure 4 shows comparisons of the sPAP and sBP during SLT in the NO, VA and VV ECMO groups. In the NO ECMO group, the sPAP significantly was elevated at the time of the main PA clamping (Fig. 4A, *P *<* *0.05). The sBP did not show a significant change at the time of starting OP, the main PA clamping and 1 h after reperfusion (Fig. 4B). In the VA ECMO group, the sPAP significantly decreased at the time of the main PA clamping (Fig. 4C, *P *<* *0.05) and the sBP showed no significant chance during SLT (Fig. 4D). In the VV ECMO group, the sPAP was significantly decreased at the time of the main PA clamping (Fig. 4E, *P *<* *0.05) and the sBP did not show significant change during SLT. In all groups, the sPAP was maintained at a low level at 1 h after reperfusion of the transplanted lung (Fig. 4A, C and E).

## DISCUSSION

We demonstrated that the PAP in the VV ECMO group during the main PA clamping on the SLT side was significantly lower compared to it at the start of OP. Unlike VA ECMO, which bypasses the heart and lung, VV ECMO does not decrease the blood flow to the lung. However, the PAP was significantly lowered after the induction of VV ECMO, as we expected. We speculate that the effect of VV ECMO on decreasing PAP was partially related to the HPV response. HPV is maximal when mixed venous oxygen saturation (SvO_2_) is normal and is decreased by either high or low SvO_2_ [7]. The large elevation of SvO_2_ during VV ECMO has been reported to potentially relieve HPV, reducing pulmonary pressure [8]. More importantly, hypercapnia is known to induce vasoconstriction of PA. Morimont *et al.* [9] showed that veno-venous extracorporeal CO_2_ removal reduced pulmonary vascular constriction related to hypercapnic acidosis and improved the pulmonary haemodynamics in a porcine acute respiratory distress syndrome model. We believe that high SvO_2_ and CO_2_ removal by VV ECMO decreases HPV and lowers the PAP during the main PA clamping on the SLT side.

The percentage of SLT among all lung transplant cases is higher in Japan, compared to other countries. Because there is a serious donor shortage in Japan, we have to choose SLT from the perspective of donor sharing [11] if the recipient does not have any infection or chylous sputum (LAM) in the lungs or PH. Hence, for instance, patients with idiopathic pulmonary fibrosis, chronic obstructive pulmonary disease, connective tissue disease-associated interstitial lung disease and bronchiolitis obliterans all undergo SLT. In each SLT case, it is important to determine the indication for intraoperative ECMO support and select the appropriate type, especially in Japan.

In the present study, the median operative durations were rather long in all groups. In Japan, the graft transportation time is long because the lung grafts are transported by public transportation, which usually results in a certain length of waiting time for the recipient’s surgery. In addition, in Asia, we have more LAM patients compared to other areas in the world and a part of those recipients (25% in our series) have past histories of pleurodesis from pneumothorax or chylothorax, which is associated with the frequent need for dissection of intrathoracic adhesion [11]. For this reason, the median intraoperative blood loss was high and the median intraoperative ECMO duration was also rather long in both the VA and VV ECMO groups. If there is a large amount of bleeding during SLT, it might be managed with a full CPB in some centres. Systemic full heparinization for CPB often causes severe bleeding in patients with intrathoracic adhesions and it may lead to re-exploration for bleeding postoperatively; therefore, we avoid CPB as much as possible.

If ECMO support is needed in a lung transplant case, VV ECMO may be considered first where possible in order to decrease the risk of bleeding complications. Large-volume transfusions of RBC and platelet are reported to be associated with various complications in lung transplant recipients in multiple studies [12]. In the present study, the amount of intraoperative RBC transfusion in the VV ECMO group tended to be smaller than that in VA ECMO. Re-exploration for bleeding was performed in no recipient in the VV ECMO group whereas 3 (13.0%) recipients needed re-exploration in the VA ECMO group.

Another important advantage of VV ECMO over VA ECMO is that we can prevent differential hypoxia, which could be caused by peripheral VA ECMO. In the phenomenon known as differential hypoxia, poorly oxygenated blood ejected into the ascending aorta from the left ventricle competes with the retrograde flow from the ECMO circuit, potentially causing myocardial and cerebral ischaemia [13]. In addition, when we weaned the patient from VA ECMO, we needed to suture the wall of the femoral vein at the decannulation sites and ligate the vascular graft sewn to the femoral artery. On the other hand, we only had to compress the decannulation sites for weaning the patient from VV ECMO, which is technically simple. VV ECMO also has the merit of accurate evaluation of the patient's tolerability to withdrawal by turning off the oxygen flow.

As a disadvantage of VA ECMO, although its incidence may be low in lung transplantation under VA ECMO support, stroke is known to occur as a complication [14]. Particularly, we should pay attention to ischaemic strokes. On the other hand, it may be one of the advantages of VA ECMO that we can easily control the blood flow of the PA on the transplant side at the time of reperfusion by reducing or increasing the VA ECMO blood flow. However, we perfuse the lung graft by slowly declamping and gradually increasing the blood flow in SLT under VV ECMO and have experienced no severe ischaemic reperfusion injury. Indeed, we demonstrated that the PGD grade was comparable between the VA and VV group.

We have changed our strategy of intraoperative ECMO support since 2015 and we assume that we could have performed more SLT cases under VV ECMO support that were performed under VA ECMO support before 2015. There are currently no established criteria for VV ECMO use when performing SLT for patients with secondary PH or decreased cardiac function. We also have to consider the perfusion ratio in the SLT side. If there is a lung offer and the recipient has a dominant perfusion ratio in the SLT side, we have to choose VA ECMO as the intraoperative support. We reported a patient with a dominant perfusion ratio in the transplant side of 89% due to a past history of lobectomy on the other side who underwent SLT under central VA ECMO [15]. Whereas the number of SLT on VV ECMO has been increasing, there are still some cases that need intraoperative VA ECMO support for SLT, and VA ECMO support is always an important option for SLT. We always perform SLT in preparation for switching the intraoperative ECMO from VV to VA or venoarteriovenous. In such a setting, the transoesophageal echocardiogram data are often important to make a decision.

### Limitations

We acknowledge that there are several limitations in the present study. First, it was a retrospective single-centre analysis with a small number of patients and thus we were not able to compare the outcome of each group accurately. Especially, the indications for SLT differ among the groups. We are planning a perspective nationwide study of intraoperative use of VV ECMO. Second, the VA ECMO group in the present study was similar to a historical control group. Therefore, it cannot be ruled out that we have improved our strategies other than VV ECMO.

## CONCLUSION

VV ECMO decreases the PAP during SLT and could therefore be a choice of ECLS during lung transplant surgery for patients, even those with PH.

## SUPPLEMENTARY MATERIAL

Supplementary material is available at *ICVTS* online.

## Funding

The authors received no specific funding for this work.

**Conflict of interest:** none declared.

## Data Availability Statement

All data are incorporated into the article and its online supplementary material.

## Author contributions

**Hisashi Oishi:** Conceptualization; Data curation; Formal analysis; Investigation; Methodology; Writing—original draft; Writing—review & editing. **Yasushi Matsuda:** Data curation; Methodology; Validation; Writing—review & editing. **Yutaka Ejima:** Data curation; Methodology; Writing—review & editing. **Hiroaki Toyama:** Data curation; Methodology; Writing—review & editing. **Takashi Hirama:** Data curation; Methodology; Writing—review & editing. **Tatsuaki Watanabe:** Data curation; Methodology; Validation; Writing—review & editing. **Yui Watanabe:** Data curation; Methodology; Writing—review & editing. Hiromichi Niikawa**:** Data curation; Methodology; Writing—review & editing. **Masafumi Noda:** Data curation; Methodology; Supervision; Validation; Writing—review& editing. **Yoshinori Okada:** Project administration; Supervision; Validation; Writing—review & editing.

## Reviewer information

Interactive CardioVascular and Thoracic Surgery thanks Sameer A. Hirji, John Dark and the other, anonymous reviewer(s) for their contribution to the peer review process of this article.

## Supplementary Material

ivac101_Supplementary_Data

## Notes

Presented at 56th Annual Congress of the Japan Society for Transplantation (web), Akita, Japan, 1–30 November 2019.

## ABBREVIATIONS

CPB: Cardiopulmonary bypass
ECLS: Extracorporeal life support
ECMO: Extracorporeal membrane oxygenation
HPV: Hypoxic pulmonary vasoconstriction
LAM: Lymphangioleiomyomatosis
NO ECMO: No use of ECMO
OP: Operation
PA: Pulmonary artery
PAP: Pulmonary arterial pressure
PGD: Primary graft dysfunction
PH: Pulmonary hypertension
RBCs: Red blood cells
sBP: Systolic blood pressure
SLT: Single lung transplantation
sPAP: Systolic PAP
sPAP: Systolic pulmonary arterial pressure
VA: Venoarterial
VV: Venovenous
