# Supplementary material for: Changes in haemodynamics during single lung transplantation under venovenous extracorporeal membrane oxygenation
Source: Interact Cardiovasc Thorac Surg. 2022 Apr 21;35(2):ivac101. doi: 10.1093/icvts/ivac101 (PMC9297505; doi:10.1093/icvts/ivac101)
Supplement: ivac101_Supplementary_Data [file ivac101_supplementary_data.docx]

**Table S1. Post-operative mortality**

|  | NO ECMO ^a^ group | VA ECMO ^b^ group | VV ECMO ^c^ group |
| --- | --- | --- | --- |
|  | (N = 13) | (N = 23) | (N = 11) |
| 30-day mortality | 0 (0%) | 0 (0%) | 0 (0%) |
| 90-day mortality | 0 (0%) | 1 (4.3%) | 1 (9.1%) |
| 1-year mortality | 0 (0%) | 1 (4.3%) | 1 (9.1%) |
| 3-year mortality | 0 (0%) | 1 (4.3%) | 1 (9.1%) |
| Values are expressed as numbers (%). ^a^ NO ECMO, no use of extracorporeal membrane oxygenation (ECMO). ^b^ VA ECMO, venoarterial ECMO. ^c^ VV ECMO, venovenous ECMO. | | | |
